# Supplementary material for: Prospective assessment of catheter-associated bacteriuria clinical presentation, epidemiology, and colonization dynamics in nursing home residents
Source: JCI Insight. 2021 Oct 8;6(19):e144775. doi: 10.1172/jci.insight.144775 (PMC8525589; doi:10.1172/jci.insight.144775)
Supplement: Supplemental data [file jciinsight-6-144775-s101.pdf]

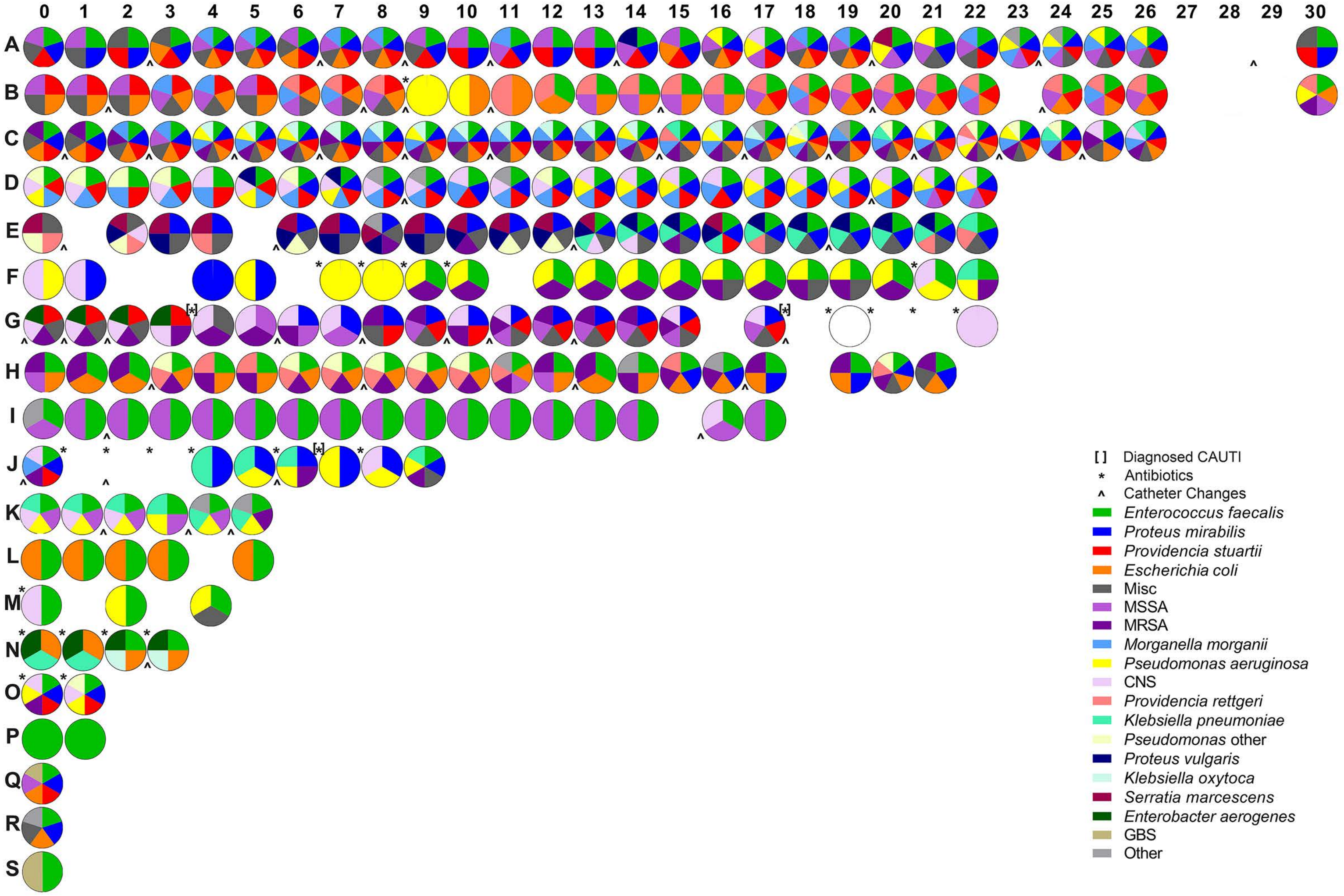

Supplemental Figure 1. Catheter-associated bacteriuria composition and dynamics. Culture results for each study participant (letters) at each study visit (numbers, starting with 0 for baseline) are presented. Empty spaces indicate study visits at which a urine specimen could not be obtained. The white circle with a black outline indicates the only culture-negative urine sample (participant G, week 19). [] caregiver-diagnosed CAUTI; *antimicrobial use; ^catheter change; Misc, Gram-positive bacteria that were PYR-negative, catalase-negative, and Streptex-negative; MSSA, methicillin-sensitive *Staphylococcus aureus*; MRSA, methicillin-resistant *Staphylococcus aureus*; CNS, coagulase-negative *Staphylococcus*; *Pseudomonas* other, Gram-negative bacteria with good identification to genus via API-20E but were not *P. aeruginosa* by PCR; GBS, Group B *Streptococcus*; Other, any Gram-negative organism that could not be identified to the species level by API-20E.

Tympanic Temperature:________ Blood Pressure:________

1st Confusion Assessment (at the very beginning of the study visit): **Time:__________**

1. Richmond Agitation-Sedation Scale (RASS) score (*see below*)			NA
2. Altered level of consciousness (RASS $\neq$ 0)	Yes	No	NA
3. Inattention: (*record answer in the space after the question*)<br>1) "Can you spell the word 'LUNCH'?"	0-1 errors	>1 error	NA

Signs and Symptoms Assessment: (*mark NA if any symptom was NOT assessed)*

4. Hypotension (blood pressure less than 90 systolic or 60 diastolic)	Yes	No	NA
5. Suprapubic pain or tenderness (palpate and verbal assessment)	Yes	No	NA
6. Costovertebral pain or tenderness (palpate and verbal assessment)	Yes	No	NA
7. Nausea (verbal assessment)	Yes	No	NA
8. Lack of appetite (verbal assessment, visual inspection of food tray)	Yes	No	NA
9. Fatigue (verbal assessment)	Yes	No	NA
10. Rigors/Shaking chills (verbal and visual assessment)	Yes	No	NA
11. Purulent discharge around catheter site (visual assessment)	Yes	No	NA
12. Gross hematuria (visual assessment)	Yes	No	NA
13. Change in functional status (qualitative week-to-week assessment)	Yes	No	NA

2nd Confusion Assessment (at the very end of the study visit): **Time:__________**

14. RASS score			NA
15. Altered level of consciousness (RASS $\neq$ 0)	Yes	No	NA
16. Inattention:<br>1) "Can you name the months from December to July?"	0-1 errors	>1 error	NA
17. Disorganized Thinking: (*record answer in the space after the question*)<br>1) "Will a stone float on water?"<br>2) "Are there fish in the sea?"<br>3) "Does one pound weigh more than two pounds?"<br>4) "Can you use a hammer to pound a nail?"<br>5) "Hold up this many fingers" (hold up two fingers)<br>"Now, do the same thing with the other hand" (do not demonstrate)	0 errors	≥1 error	NA

Richmond Agitation-Sedation Scale (RASS)

Score	Term	Description	
+4	Combative	Overtly combative, violent, immediate danger to staff	
+3	Very agitated	Pulls or removes tube(s) or catheter(s); aggressive	
+2	Agitated	Frequent nonpurposeful movement, fights ventilator	
+1	Restless	Anxious but movements not aggressive or vigorous	
0	Alert and calm		
−1	Drowsy	Not fully alert, but has sustained awakening (eye opening/eye contact) to voice (>10 seconds)	Verbal stimulation
−2	Light sedation	Briefly awakens with eye contact to voice (<10 seconds)	Verbal stimulation
−3	Moderate sedation	Movement or eye opening to voice (but no eye contact)	Verbal stimulation
−4	Deep sedation	No response to voice, but movement or eye opening to physical stimulation	Physical stimulation
−5	Unarousable	No response to voice or physical stimulation	Physical stimulation

Procedure for RASS Assessment

1. Observe patient
   - Patient is alert, restless, or agitated. — Score 0 to +4
2. If not alert, state patient's name and say to open eyes and look at speaker.
   - Patient awakens with sustained eye opening and eye contact. — Score −1
   - Patient awakens with eye opening and eye contact, but not sustained. — Score −2
   - Patient has any movement in response to voice but no eye contact. — Score −3
3. When no response to verbal stimulation, physically stimulate patient by shaking shoulder and/or rubbing sternum.
   - Patient has any movement to physical stimulation. — Score −4
   - Patient has no response to any stimulation. — Score −5

Supplemental Figure 2. Clinical sign and symptom assessment tool. This worksheet was completed by a licensed practicing nurse at each study visit to prospectively assess possible signs and symptoms of infection, in addition to information collected by chart review.

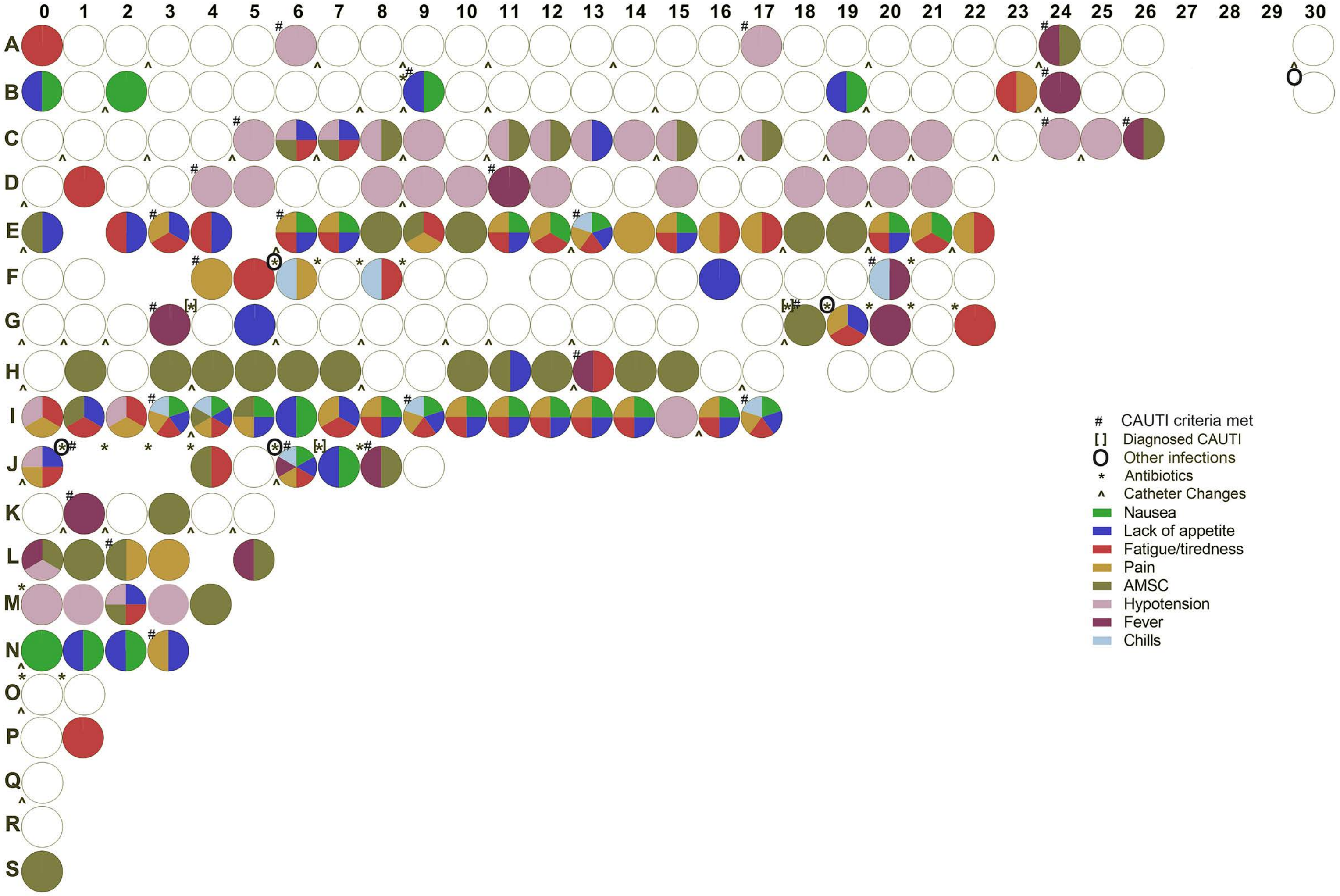

Supplemental Figure 3. Clinical presentation of catheter-associated bacteriuria. Potential signs and symptoms of infection for each study participant (letters) at each study visit (numbers, starting with 0 for baseline) are presented. Empty spaces indicate study visits at which signs and symptoms of infection could not be assessed, and white circles with black outlines indicate the absence of signs and symptoms at a particular visit. #study visits at which the NHSN CAUTI surveillance criteria would be met if urine specimens with ≥2 bacterial species were included; [] caregiver-diagnosed CAUTI; O caregiver-diagnosed infections other than CAUTI; *antimicrobial use; ^catheter change.

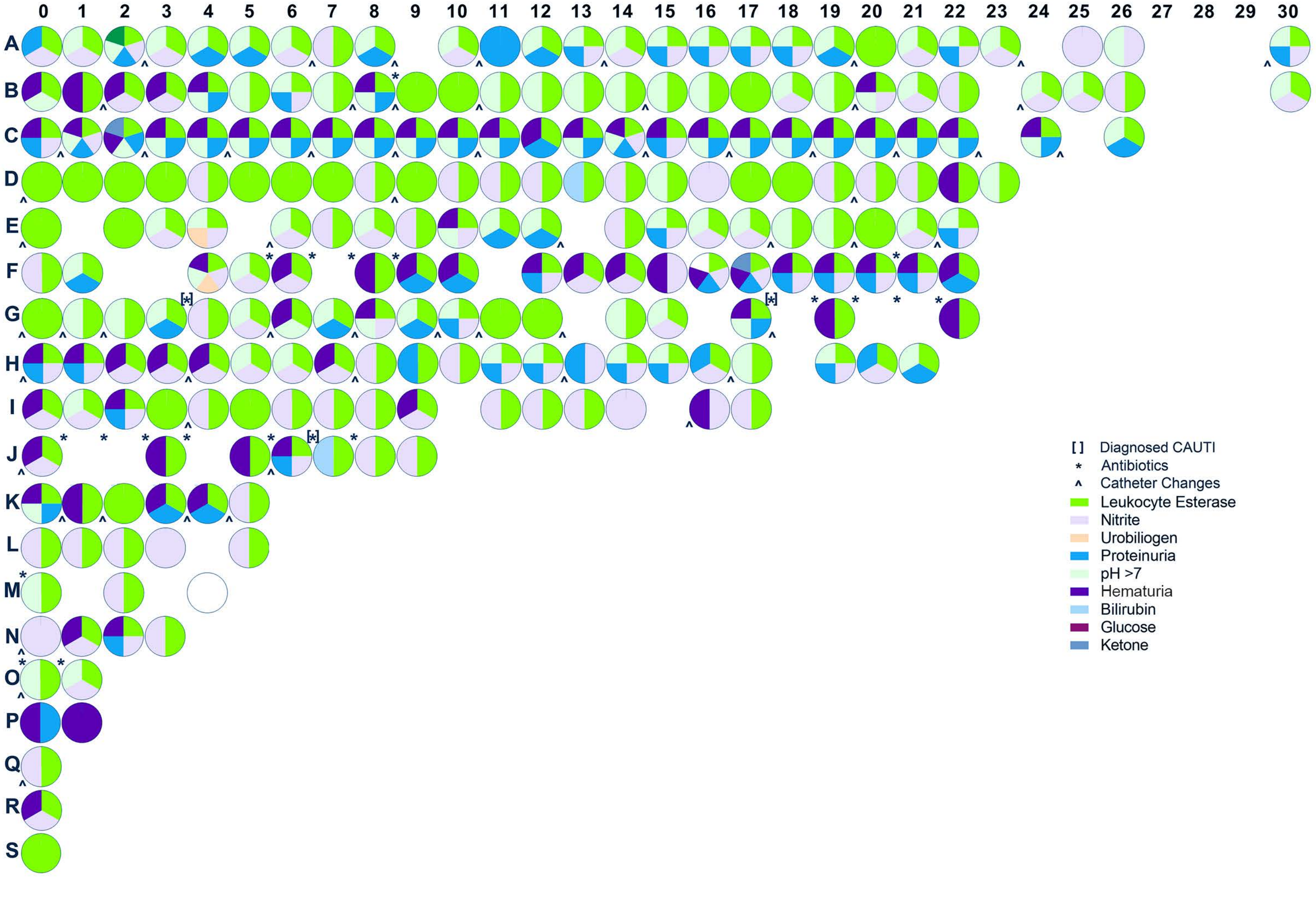

Supplemental Figure 4. Urinalysis of catheter-associated bacteriuria. Urinalysis test strip results for each study participant (letters) at each study visit (numbers, starting with 0 for baseline) are presented. Empty spaces indicate study visits at which urinalysis could not be performed, and white circles with black outlines indicate the absence of any abnormal urinalysis findings at a particular visit. [] caregiver-diagnosed CAUTI; *antimicrobial use; ^catheter change.

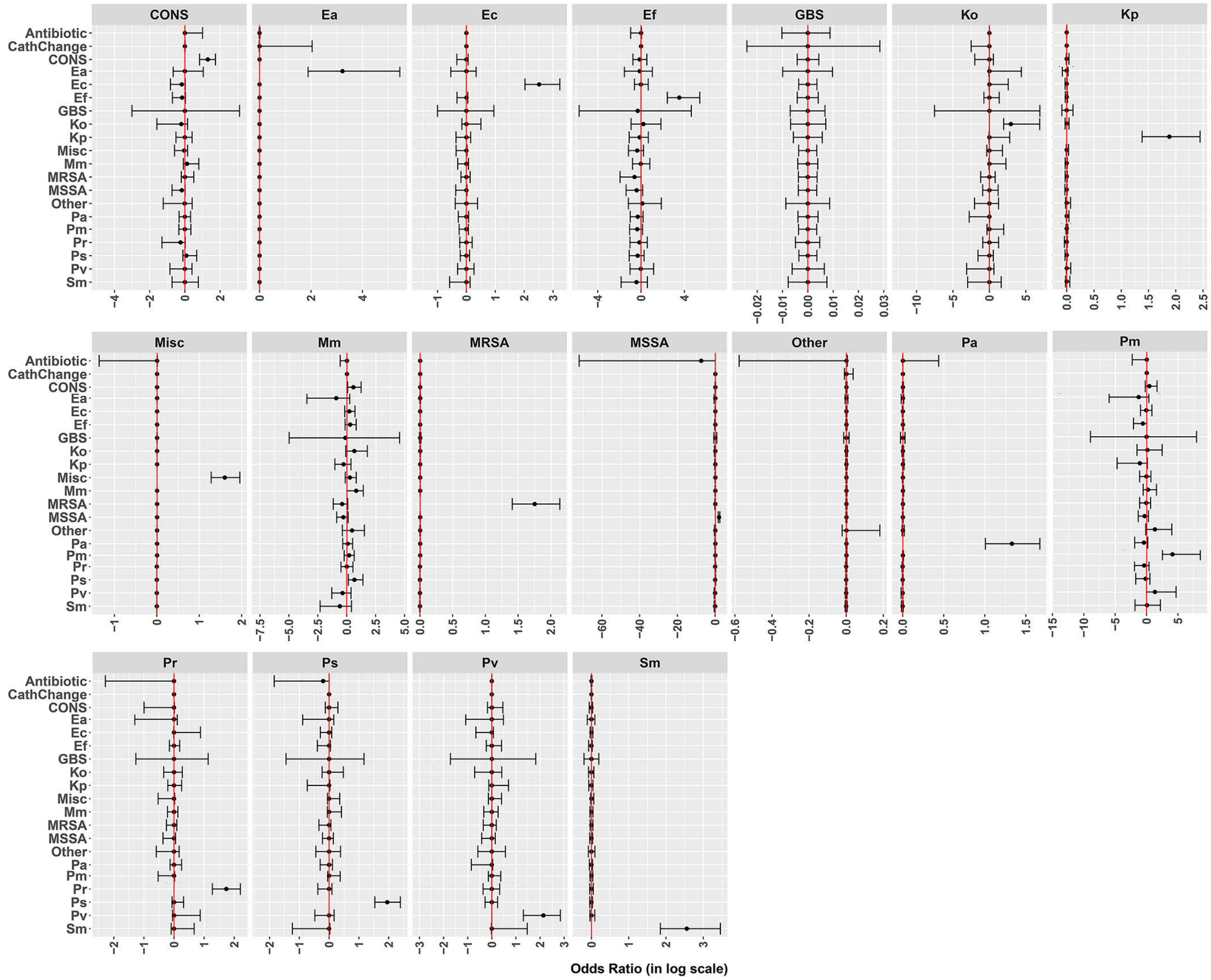

Supplemental Figure 5. Bayesian multilevel longitudinal models were used to analyze the contribution of several parameters (antibiotic use, catheter changes, and colonization by specific microbes) at a given study visit to the likelihood of a specific microbe being present at the following study visit. Each panel displays the contribution of each parameter on the left axis to the likelihood of colonization by the organism listed at the top of the panel. Forest plots display the posterior medians and 95% credible intervals (computed through 0.025th and 0.975th posterior quantiles). Misc, Gram-positive bacteria that were PYR-negative, catalase-negative, and Streptex-negative; MSSA, methicillin-sensitive *Staphylococcus aureus*; MRSA, methicillin-resistant *Staphylococcus aureus*; CONS, coagulase-negative *Staphylococcus*; GBS, Group B *Streptococcus*; Other, any Gram-negative organism that could not be identified to the species level by API-20E.

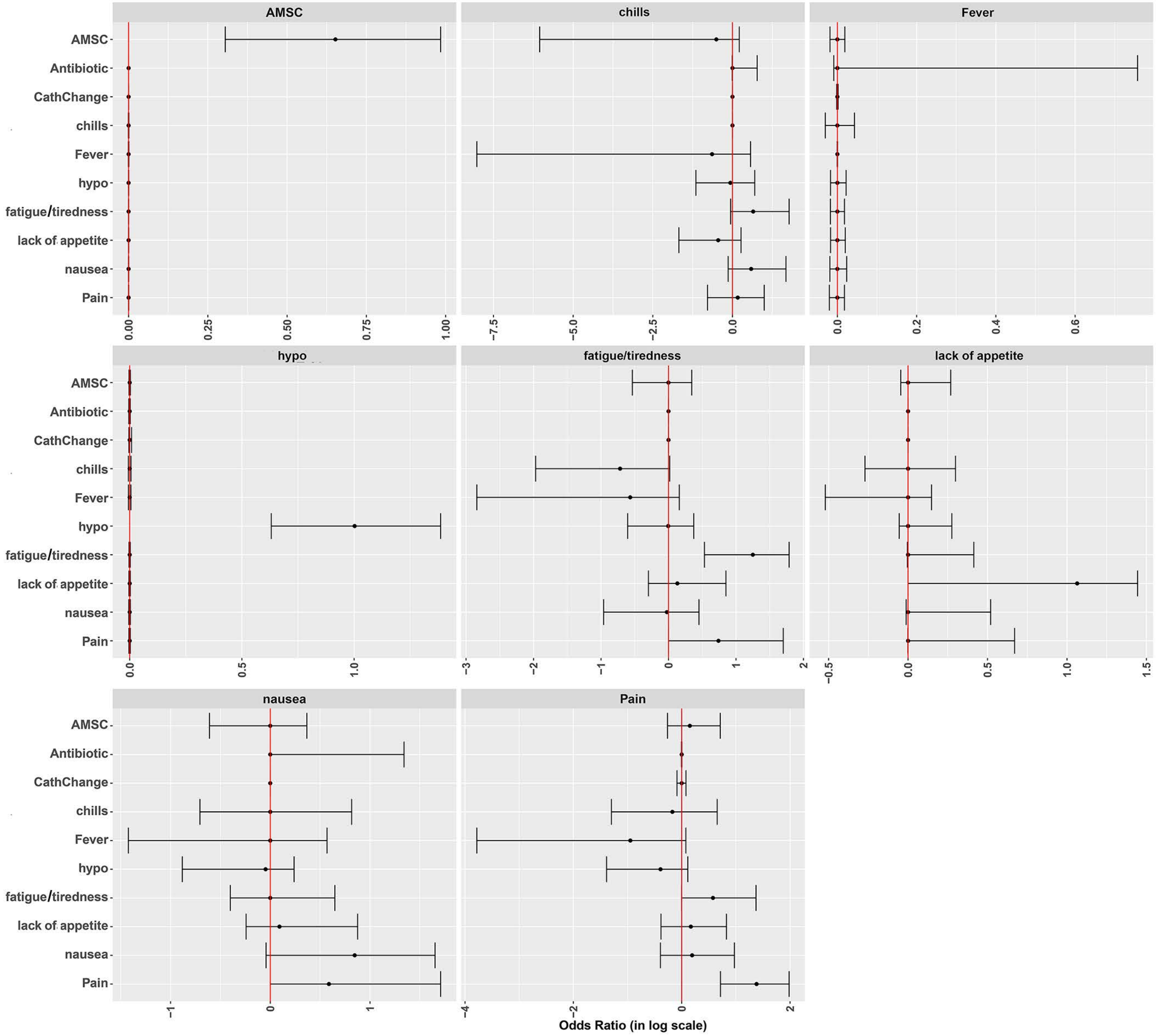

Supplemental Figure 6. Bayesian multilevel longitudinal models were used to analyze the contribution of several parameters (antibiotic use, catheter changes, and possible signs and symptoms of infection) at a given study visit to the likelihood of a specific sign or symptom of infection being present at the following study visit. Each panel displays the contribution of each parameter on the left axis to the likelihood of the sign or symptom listed at the top of the panel. Forest plots display the posterior medians and 95% credible intervals (computed through 0.025th and 0.975th posterior quantiles).
