# Supplementary material for: Prospective assessment of catheter-associated bacteriuria clinical presentation, epidemiology, and colonization dynamics in nursing home residents
Source: JCI Insight. 2021 Oct 8;6(19):e144775. doi: 10.1172/jci.insight.144775 (PMC8525589; doi:10.1172/jci.insight.144775)
Supplement: ICMJE disclosure forms [file jciinsight-6-144775-s102.pdf]

ICMJE DISCLOSURE FORM

Date:___Aug 26, 2021____
Your Name:____Chelsie Armbruster____
Manuscript Title:_Prospective assessment of catheter-associated bacteriuria in nursing home residents: clinical presentation, epidemiology, and colonization dynamics____
Manuscript number (if known):__144775-INS-CMED-1____

In the interest of transparency, we ask you to disclose all relationships/activities/interests listed below that are related to the content of your manuscript. "Related" means any relation with for-profit or not-for-profit third parties whose interests may be affected by the content of the manuscript. Disclosure represents a commitment to transparency and does not necessarily indicate a bias. If you are in doubt about whether to list a relationship/activity/interest, it is preferable that you do so.

The following questions apply to the author's relationships/activities/interests as they relate to the current manuscript only.

The author's relationships/activities/interests should be defined broadly. For example, if your manuscript pertains to the epidemiology of hypertension, you should declare all relationships with manufacturers of antihypertensive medication, even if that medication is not mentioned in the manuscript.

In item #1 below, report all support for the work reported in this manuscript without time limit. For all other items, the time frame for disclosure is the past 36 months.

		**Name all entities with whom you have this relationship or indicate none (add rows as needed)**	**Specifications/Comments (e.g., if payments were made to you or to your institution)**
		**Time frame: Since the initial planning of the work**	
1	All support for the present manuscript (e.g., funding, provision of study materials, medical writing, article processing charges, etc.) **No time limit for this item.**	____ None<br>NIH	R00 DK105205, R01 DK123158, UL1 TR001412
		**Time frame: past 36 months**	
2	Grants or contracts from any entity (if not indicated in item #1 above).	__x__ None	
3	Royalties or licenses	__x__ None	

4	Consulting fees	__x__ None	
5	Payment or honoraria for lectures, presentations, speakers bureaus, manuscript writing or educational events	____ None<br>Western New York branch, American Society for Microbiology	Honorarium for presentation at annual meeting
6	Payment for expert testimony	__x__ None	
7	Support for attending meetings and/or travel	__x__ None	
8	Patents planned, issued or pending	__x__ None	
9	Participation on a Data Safety Monitoring Board or Advisory Board	__x__ None	
10	Leadership or fiduciary role in other board, society, committee or advocacy group, paid or unpaid	__x__ None	
11	Stock or stock options	__x__ None	
12	Receipt of equipment, materials, drugs, medical writing, gifts or other services	__x__ None	
13	Other financial or non-financial interests	__x__ None	

Please place an "X" next to the following statement to indicate your agreement:

_x__ I certify that I have answered every question and have not altered the wording of any of the questions on this form.

ICMJE DISCLOSURE FORM

Date:___Aug 26, 2021____
Your Name:____Aimee Brauer____
Manuscript Title:_Prospective assessment of catheter-associated bacteriuria in nursing home residents: clinical presentation, epidemiology, and colonization dynamics____
Manuscript number (if known):__144775-INS-CMED-1____

In the interest of transparency, we ask you to disclose all relationships/activities/interests listed below that are related to the content of your manuscript. "Related" means any relation with for-profit or not-for-profit third parties whose interests may be affected by the content of the manuscript. Disclosure represents a commitment to transparency and does not necessarily indicate a bias. If you are in doubt about whether to list a relationship/activity/interest, it is preferable that you do so.

The following questions apply to the author's relationships/activities/interests as they relate to the current manuscript only.

The author's relationships/activities/interests should be defined broadly. For example, if your manuscript pertains to the epidemiology of hypertension, you should declare all relationships with manufacturers of antihypertensive medication, even if that medication is not mentioned in the manuscript.

In item #1 below, report all support for the work reported in this manuscript without time limit. For all other items, the time frame for disclosure is the past 36 months.

		**Name all entities with whom you have this relationship or indicate none (add rows as needed)**	**Specifications/Comments (e.g., if payments were made to you or to your institution)**
		**Time frame: Since the initial planning of the work**	
1	All support for the present manuscript (e.g., funding, provision of study materials, medical writing, article processing charges, etc.) **No time limit for this item.**	__x__ None	
		**Time frame: past 36 months**	
2	Grants or contracts from any entity (if not indicated in item #1 above).	__x__ None	
3	Royalties or licenses	__x__ None	

4	Consulting fees	__x__ None	
5	Payment or honoraria for lectures, presentations, speakers bureaus, manuscript writing or educational events	__x__ None	
6	Payment for expert testimony	__x__ None	
7	Support for attending meetings and/or travel	__x__ None	
8	Patents planned, issued or pending	__x__ None	
9	Participation on a Data Safety Monitoring Board or Advisory Board	__x__ None	
10	Leadership or fiduciary role in other board, society, committee or advocacy group, paid or unpaid	__x__ None	
11	Stock or stock options	__x__ None	
12	Receipt of equipment, materials, drugs, medical writing, gifts or other services	__x__ None	
13	Other financial or non-financial interests	__x__ None	

Please place an "X" next to the following statement to indicate your agreement:

_x__ I certify that I have answered every question and have not altered the wording of any of the questions on this form.

ICMJE DISCLOSURE FORM

Date:___Aug 26, 2021____
Your Name:____Monica Humby____
Manuscript Title:_Prospective assessment of catheter-associated bacteriuria in nursing home residents: clinical presentation, epidemiology, and colonization dynamics____
Manuscript number (if known):__144775-INS-CMED-1____

In the interest of transparency, we ask you to disclose all relationships/activities/interests listed below that are related to the content of your manuscript. "Related" means any relation with for-profit or not-for-profit third parties whose interests may be affected by the content of the manuscript. Disclosure represents a commitment to transparency and does not necessarily indicate a bias. If you are in doubt about whether to list a relationship/activity/interest, it is preferable that you do so.

The following questions apply to the author's relationships/activities/interests as they relate to the <u>current manuscript only</u>.

The author's relationships/activities/interests should be <u>defined broadly</u>. For example, if your manuscript pertains to the epidemiology of hypertension, you should declare all relationships with manufacturers of antihypertensive medication, even if that medication is not mentioned in the manuscript.

In item #1 below, report all support for the work reported in this manuscript without time limit. For all other items, the time frame for disclosure is the past 36 months.

		**Name all entities with whom you have this relationship or indicate none (add rows as needed)**	**Specifications/Comments (e.g., if payments were made to you or to your institution)**
		**Time frame: Since the initial planning of the work**	
1	All support for the present manuscript (e.g., funding, provision of study materials, medical writing, article processing charges, etc.) **No time limit for this item.**	__x__ None	
		**Time frame: past 36 months**	
2	Grants or contracts from any entity (if not indicated in item #1 above).	__x__ None	
3	Royalties or licenses	__x__ None	

4	Consulting fees	__x__ None	
5	Payment or honoraria for lectures, presentations, speakers bureaus, manuscript writing or educational events	__x__ None	
6	Payment for expert testimony	__x__ None	
7	Support for attending meetings and/or travel	__x__ None	
8	Patents planned, issued or pending	__x__ None	
9	Participation on a Data Safety Monitoring Board or Advisory Board	__x__ None	
10	Leadership or fiduciary role in other board, society, committee or advocacy group, paid or unpaid	__x__ None	
11	Stock or stock options	__x__ None	
12	Receipt of equipment, materials, drugs, medical writing, gifts or other services	__x__ None	
13	Other financial or non-financial interests	__x__ None	

Please place an "X" next to the following statement to indicate your agreement:

_x__ I certify that I have answered every question and have not altered the wording of any of the questions on this form.

ICMJE DISCLOSURE FORM

Date:___Aug 26, 2021____
Your Name:____Jiahui Shao____
Manuscript Title:_Prospective assessment of catheter-associated bacteriuria in nursing home residents: clinical presentation, epidemiology, and colonization dynamics____
Manuscript number (if known):__144775-INS-CMED-1____

In the interest of transparency, we ask you to disclose all relationships/activities/interests listed below that are related to the content of your manuscript. "Related" means any relation with for-profit or not-for-profit third parties whose interests may be affected by the content of the manuscript. Disclosure represents a commitment to transparency and does not necessarily indicate a bias. If you are in doubt about whether to list a relationship/activity/interest, it is preferable that you do so.

The following questions apply to the author's relationships/activities/interests as they relate to the current manuscript only.

The author's relationships/activities/interests should be defined broadly. For example, if your manuscript pertains to the epidemiology of hypertension, you should declare all relationships with manufacturers of antihypertensive medication, even if that medication is not mentioned in the manuscript.

In item #1 below, report all support for the work reported in this manuscript without time limit. For all other items, the time frame for disclosure is the past 36 months.

		Name all entities with whom you have this relationship or indicate none (add rows as needed)	Specifications/Comments (e.g., if payments were made to you or to your institution)
		**Time frame: Since the initial planning of the work**	
1	All support for the present manuscript (e.g., funding, provision of study materials, medical writing, article processing charges, etc.) **No time limit for this item.**	__x__ None	
		**Time frame: past 36 months**	
2	Grants or contracts from any entity (if not indicated in item #1 above).	__x__ None	
3	Royalties or licenses	__x__ None	

4	Consulting fees	__x__ None	
5	Payment or honoraria for lectures, presentations, speakers bureaus, manuscript writing or educational events	__x__ None	
6	Payment for expert testimony	__x__ None	
7	Support for attending meetings and/or travel	__x__ None	
8	Patents planned, issued or pending	__x__ None	
9	Participation on a Data Safety Monitoring Board or Advisory Board	__x__ None	
10	Leadership or fiduciary role in other board, society, committee or advocacy group, paid or unpaid	__x__ None	
11	Stock or stock options	__x__ None	
12	Receipt of equipment, materials, drugs, medical writing, gifts or other services	__x__ None	
13	Other financial or non-financial interests	__x__ None	

Please place an "X" next to the following statement to indicate your agreement:

_x__ I certify that I have answered every question and have not altered the wording of any of the questions on this form.

ICMJE DISCLOSURE FORM

Date:___Aug 26, 2021____
Your Name:____Saptarshi Chakraborty____
Manuscript Title:_Prospective assessment of catheter-associated bacteriuria in nursing home residents: clinical presentation, epidemiology, and colonization dynamics____
Manuscript number (if known):__144775-INS-CMED-1____

In the interest of transparency, we ask you to disclose all relationships/activities/interests listed below that are related to the content of your manuscript. "Related" means any relation with for-profit or not-for-profit third parties whose interests may be affected by the content of the manuscript. Disclosure represents a commitment to transparency and does not necessarily indicate a bias. If you are in doubt about whether to list a relationship/activity/interest, it is preferable that you do so.

The following questions apply to the author's relationships/activities/interests as they relate to the current manuscript only.

The author's relationships/activities/interests should be defined broadly. For example, if your manuscript pertains to the epidemiology of hypertension, you should declare all relationships with manufacturers of antihypertensive medication, even if that medication is not mentioned in the manuscript.

In item #1 below, report all support for the work reported in this manuscript without time limit. For all other items, the time frame for disclosure is the past 36 months.

		**Name all entities with whom you have this relationship or indicate none (add rows as needed)**	**Specifications/Comments (e.g., if payments were made to you or to your institution)**
		**Time frame: Since the initial planning of the work**	
1	All support for the present manuscript (e.g., funding, provision of study materials, medical writing, article processing charges, etc.) **No time limit for this item.**	__x__ None	
		**Time frame: past 36 months**	
2	Grants or contracts from any entity (if not indicated in item #1 above).	__x__ None	
3	Royalties or licenses	__x__ None	

4	Consulting fees	__x__ None	
5	Payment or honoraria for lectures, presentations, speakers bureaus, manuscript writing or educational events	__x__ None	
6	Payment for expert testimony	__x__ None	
7	Support for attending meetings and/or travel	__x__ None	
8	Patents planned, issued or pending	__x__ None	
9	Participation on a Data Safety Monitoring Board or Advisory Board	__x__ None	
10	Leadership or fiduciary role in other board, society, committee or advocacy group, paid or unpaid	__x__ None	
11	Stock or stock options	__x__ None	
12	Receipt of equipment, materials, drugs, medical writing, gifts or other services	__x__ None	
13	Other financial or non-financial interests	__x__ None	

Please place an "X" next to the following statement to indicate your agreement:

_x__ I certify that I have answered every question and have not altered the wording of any of the questions on this form.

STROBE Statement—Checklist of items that should be included in reports of ***cohort studies***

	Item No	Recommendation
**Title and abstract**	1	(*a*) Indicate the study's design with a commonly used term in the title or the abstract
		(*b*) Provide in the abstract an informative and balanced summary of what was done and what was found
**Introduction**		
Background/rationale	2	Explain the scientific background and rationale for the investigation being reported
Objectives	3	State specific objectives, including any prespecified hypotheses
**Methods**		
Study design	4	Present key elements of study design early in the paper
Setting	5	Describe the setting, locations, and relevant dates, including periods of recruitment, exposure, follow-up, and data collection
Participants	6	(*a*) Give the eligibility criteria, and the sources and methods of selection of participants. Describe methods of follow-up
		(*b*) For matched studies, give matching criteria and number of exposed and unexposed
Variables	7	Clearly define all outcomes, exposures, predictors, potential confounders, and effect modifiers. Give diagnostic criteria, if applicable
Data sources/ measurement	8*	For each variable of interest, give sources of data and details of methods of assessment (measurement). Describe comparability of assessment methods if there is more than one group
Bias	9	Describe any efforts to address potential sources of bias
Study size	10	Explain how the study size was arrived at
Quantitative variables	11	Explain how quantitative variables were handled in the analyses. If applicable, describe which groupings were chosen and why
Statistical methods	12	(*a*) Describe all statistical methods, including those used to control for confounding
		(*b*) Describe any methods used to examine subgroups and interactions
		(*c*) Explain how missing data were addressed
		(*d*) If applicable, explain how loss to follow-up was addressed
		(*e*) Describe any sensitivity analyses
**Results**		
Participants	13*	(a) Report numbers of individuals at each stage of study—eg numbers potentially eligible, examined for eligibility, confirmed eligible, included in the study, completing follow-up, and analysed
		(b) Give reasons for non-participation at each stage
		(c) Consider use of a flow diagram
Descriptive data	14*	(a) Give characteristics of study participants (eg demographic, clinical, social) and information on exposures and potential confounders
		(b) Indicate number of participants with missing data for each variable of interest
		(c) Summarise follow-up time (eg, average and total amount)
Outcome data	15*	Report numbers of outcome events or summary measures over time
Main results	16	(*a*) Give unadjusted estimates and, if applicable, confounder-adjusted estimates and their precision (eg, 95% confidence interval). Make clear which confounders were adjusted for and why they were included
		(*b*) Report category boundaries when continuous variables were categorized
		(*c*) If relevant, consider translating estimates of relative risk into absolute risk for a meaningful time period

Other analyses	17	Report other analyses done—eg analyses of subgroups and interactions, and sensitivity analyses
**Discussion**		
Key results	18	Summarise key results with reference to study objectives
Limitations	19	Discuss limitations of the study, taking into account sources of potential bias or imprecision. Discuss both direction and magnitude of any potential bias
Interpretation	20	Give a cautious overall interpretation of results considering objectives, limitations, multiplicity of analyses, results from similar studies, and other relevant evidence
Generalisability	21	Discuss the generalisability (external validity) of the study results
**Other information**		
Funding	22	Give the source of funding and the role of the funders for the present study and, if applicable, for the original study on which the present article is based

*Give information separately for exposed and unexposed groups.

Note: An Explanation and Elaboration article discusses each checklist item and gives methodological background and published examples of transparent reporting. The STROBE checklist is best used in conjunction with this article (freely available on the Web sites of PLoS Medicine at http://www.plosmedicine.org/, Annals of Internal Medicine at http://www.annals.org/, and Epidemiology at http://www.epidem.com/). Information on the STROBE Initiative is available at http://www.strobe-statement.org.
